# Assessing the Quality of Serological Testing in the COVID-19 Pandemic: Results of a European External Quality Assessment (EQA) Scheme for Anti-SARS-CoV-2 Antibody Detection

**DOI:** 10.1128/JCM.00559-21

**Published:** 2021-08-18

**Authors:** Volker Ast, Victor Costina, Romy Eichner, Anna Bode, Sihem Aida, Catharina Gerhards, Margot Thiaucourt, Gerhard Dobler, Wolf-Jochen Geilenkeuser, Roman Wölfel, Michael Neumaier, Verena Haselmann

**Affiliations:** a Department of Clinical Chemistry, University Medical Centre Mannheim, Medical Faculty Mannheim of the University of Heidelberg, Mannheim, Germany; b Reference-Institute for Bioanalytics, German Society for Clinical Chemistry and Laboratory Medicine (DGKL), Bonn, Germany; c Bundeswehr Institute of Microbiology, Munich, Germany; d German Center for Infection Research (DZIF), Partner Site Munich, Germany; Cepheid

**Keywords:** SARS-CoV-2, test performance, external quality assessment scheme, ring trial, proficiency testing, COVID-19, anti-SARS-CoV-2 antibodies, EQA, immunoassays

## Abstract

External quality assessment (EQA) is a key instrument for achieving harmonization, and thus a high quality, of diagnostic procedures. As reliable test results are crucial for accurate assessment of SARS-CoV-2 infection prevalence, vaccine response, and immunity, and thus for successful management of the ongoing COVID-19 pandemic, the Reference Institute for Bioanalytics (RfB) was the first EQA provider to offer an open scheme for anti-SARS-CoV-2 antibody detection. The main objectives of this EQA were (i) to gain insights into the current diagnostic landscape and the performance of serological tests in Europe and (ii) to provide recommendations for diagnostic improvements. Within the EQA, a blinded panel of precharacterized human serum samples with variable anti-SARS-CoV-2 antibody titers was provided for detection of anti-SARS-CoV-2 IgG, IgA, and IgM antibodies. Across the three distribution rounds in 2020, 284 laboratories from 22 countries reported a total of 3,744 results for anti-SARS-CoV-2 antibody detection using more than 24 different assays for IgG. Overall, 97/3,004 results were false for anti-SARS-CoV-2 IgG, 88/248 for IgA, and 34/124 for IgM. Regarding diagnostic sensitivity and specificity, substantial differences were found between the different assays used, as well as between certified and noncertified tests. For cutoff samples, a drop in the diagnostic sensitivity to 46.3% and high interlaboratory variability were observed. In general, this EQA highlights the current variability of anti-SARS-CoV-2 antibody detection, technical limitations with respect to cutoff samples, and the lack of harmonization of testing procedures. Recommendations are provided to help laboratories and manufacturers further improve the quality of anti-SARS-CoV-2 serological diagnostics.

## INTRODUCTION

Accurate and reliable diagnosis of severe acute respiratory syndrome coronavirus 2 (SARS-CoV-2) infection and acute respiratory disease caused by SARS-CoV-2, termed coronavirus disease 2019 (COVID-19), is of paramount importance for successful management of the current pandemic.

Following the emergence of SARS-CoV-2, molecular and serological diagnostic strategies have been developed rapidly by numerous companies and implemented on a large scale at a fast pace, facilitated by emergency use authorization (EUA) (1–4). While diagnosis of acute infection relies on reverse transcription-quantitative PCR (qRT-PCR)-based viral detection in respiratory material (5), serological testing is recommended to retrospectively assess seroprevalence rates, as a diagnostic aid for patients with negative qRT-PCR results, to determine vaccine response and duration of immunity, and to identify suitable convalescent blood donors (6–8). Serological assays can detect IgM, IgG, IgA, or total antibodies directed against the SARS-CoV-2 nucleocapsid protein, spike protein, or receptor binding domain. They are based on various assay formats, such as enzyme-linked immunosorbent assays (ELISAs), chemiluminescent immunoassays (CLIAs), or chemiluminescent microparticle assays (CMIAs) (8, 9). Due to this rapidly growing, diverse diagnostic landscape of serological tests, accompanied by publications of heterogeneous or questionable assay performance (10, 11), assurance of and improvement in diagnostic quality are of utmost importance in the context of the global health situation.

Proficiency testing (PT) is one of the main tools of infectious diseases diagnostic quality assessment (12). PT/external quality assessment (EQA) is a key instrument for independently assessing the diagnostic performance of laboratories and of the methods currently in use, for identifying shortcomings, and for contributing to harmonizing and standardizing of diagnostic procedures by providing recommendations (13–15). In such an EQA scheme, a blinded panel, regularly comprising 2 to 10 negative and positive samples, is distributed by an accredited EQA provider to the participating laboratories, which must use their standard operation procedures to analyze and report the results for evaluation and certification within a predetermined time frame (15).

In the case of anti-SARS-CoV-2 serological testing, the Reference Institute for Bioanalytics (RfB) was the first EQA provider to conduct a pilot scheme in April 2020 (16). After the feasibility of the scheme’s design was proven, the EQA was opened at the international level, and three distribution rounds were scheduled in 2020 (17). In this report, we present the outcome of these distribution rounds with the aims of (i) providing an overview of the current anti-SARS-CoV-2 serological landscape, (ii) offering insights into diagnostic performance, and (iii) making recommendations for further improvements.

## MATERIALS AND METHODS

### EQA design.

The EQA scheme for SARS-CoV-2 antibody detection conducted by the RfB is an open EQA that was announced via the RfB program and its website (https://www.rfb.bio/). The RfB has an accreditation according to DIN EN ISO/IEC 17043:2010 as an EQA provider. Each of the three schemes conducted in 2020 (May, August, October) consisted of one panel of human serum samples for the analysis of anti-SARS-CoV-2 IgG, anti-SARS-CoV-2 IgM, or anti-SARS-CoV-2 IgA antibodies. Each panel consisted of four precharacterized, pseudonymized serum samples from voluntary donors. The positive samples comprised patient sera with various anti-SARS-CoV-2 antibody titers. All patients were recruited at University Medical Centre Mannheim, Germany. The study was approved by the Institutional Review Board, and informed written consent was obtained from each subject prior to sample collection, analysis, and dispatch. The study was conducted in accordance with the Declaration of Helsinki. The medical history of each subject was recorded with standardized questionnaires, and detailed information is provided in the supplemental material.

The samples were distributed by the RfB at ambient temperature, in accordance with sample stability as assessed in validation studies (data not shown) and other serological EQA schemes. Each participating laboratory received a 600-μl blind aliquot of each sample for COVID-19 antibody detection. Each sample dispatch was accompanied by a covering letter giving basic instructions for specimen handling and reporting of results. Participants were asked to use their standard operation procedures to determine the anti-SARS-CoV-2 antibody class and to report qualitative results (positive, negative, or borderline) within 5 days. All reports were assessed by the RfB scheme organizers. The following criteria were chosen as minimum requirements for successful participation: (i) correct identification of all samples provided with respect to the antibody class tested and (ii) results reported for all samples provided. A general report summarizing the statistics and final results was sent to all participating laboratories, together with a certificate for anti-SARS-CoV-2 serological testing for correctly determined Ig class.

### Preparation and characterization of EQA samples by the RfB.

The EQA samples were prepared according to standard operation procedures, as described in the following paragraphs and depicted in Fig. S1.

After blood draw, serum samples were stored at ambient temperature for at least 1 h to allow appropriate clotting. Clotted samples were centrifuged for 10 min at 2,000 × *g* and 18°C within 4 h after sample collection. Then, serum was pooled and divided into 600-μl aliquots (at least 10 aliquots for precharacterization), and finally, the serum pool and the aliquots were stored at −80°C. One day before shipment, the remaining serum pool was thawed and divided into 600-μl aliquots. The RfB scheme organizers’ laboratories (Institute of Clinical Chemistry, UMM, Mannheim and Bundeswehr Institute of Microbiology, Munich) tested at least 3 aliquots and the pool of each specimen for anti-SARS-CoV-2-specific IgG, IgM, or IgA antibodies, as well as for virus-neutralizing antibodies, prior to sample dispatch. The absolute results (ratios/cutoff indexes [COIs]) are summarized in Table 1. All results were discussed by a panel of experts, and based on the results and patients’ clinics, a consensus/target value was assigned to each sample and antibody class.

**TABLE 1:** Sample characterization by RfB prior to dispatch to the participants^*a*^

Sample no.	EQA scheme no.	VNT titer	IgG characteristics						IgA characteristics			IgM characteristics		Medical report
			Target value	Laboratory 1				Laboratory 2	Target value	Laboratory 1	Laboratory 2	Target value	Laboratory 1	
				Roche Elecsys anti-N (COI)^*c*^	Roche Elecsys anti-S (U/ml)^*d*^	Euroimmun anti-S(ratio)^*e*^	Epitope anti-N(ratio)^*f*^	Euroimmun anti-S(ratio)^*g*^		Euroimmun anti-S(ratio)^*h*^	Euroimmun anti-S(ratio)^*i*^		Epitope anti-N(ratio)^*j*^	
1	1	1:40^*b*^	Positive	2.86	NA	1.41	0.41	1.1	ND	ND	0.97	Negative	0.11	SARS-CoV-2 positive-tested, symptomatic patient. Blood sampling 34 days after symptom onset.
2	1	1:10	Positive	34.64	NA	2.91	0.44	2.11	ND	ND	0.22	Negative	0.12	SARS-CoV-2 positive-tested, asymptomatic patient. Blood sampling 39 days after symptom onset.
3	1	<1:10	Negative	0.07	NA	0.49	0.19	0.32	ND	ND	0.36	Negative	0.13	SARS-CoV-2-negative patient.
4	1	<1:10	Negative	0.48	NA	0.75	0.22	0.51	ND	ND	0.49	Negative	0.13	SARS-CoV-2-positive-tested, oligosymptomatic patient. Blood sampling 26 days after symptom onset.
5	2	<1:10	Positive	5.01	NA	0.84	ND	0.84	Negative	0.7	0.54	ND	ND	SARS-CoV-2-postitive-tested, symptomatic patient. Blood sampling 37 days after symptom onset.
6	2	<1:10	Negative	0.05	NA	0.35	ND	0.2	Negative	0.57	0.27	ND	ND	SARS-CoV-2 negative patient.
7	2	<1:10	Negative	0.06	NA	0.48	ND	0.26	Negative	1.63	0.59	ND	ND	SARS-CoV-2 negative patient pool.
8	2	1:20	Positive	80.46	NA	5.75	ND	7.86	Positive	2.44	1.7	ND	ND	SARS-CoV-2-positive-tested, oligosymptomatic patient. Blood sampling 59 days after symptom onset.
9	3	1:20	Positive	15.02	53.05	2.36	ND	3.3	Positive	1.19	2.3	ND	ND	SARS-CoV-2 positive-tested, symptomatic patient. Blood sampling 85 days after symptom onset.
10	3	<1:10	Positive	0.89	9.09	1.16	ND	1.36	Positive	1.89	2.84	ND	ND	SARS-CoV-2-positive-tested, oligosymptomatic patient. Blood sampling 32 days after symptom onset.
11	3	1:40	Positive	12.23	15.49	6.05	ND	9.56	Positive	3.69	5.37	ND	ND	SARS-CoV-2-positive-tested, oligosymptomatic patient. Blood sampling 47 days after symptom onset.
12	3	<1:10	Negative	0.06	0.4	0.40	ND	0.28	Negative	0.28	0.24	ND	ND	SARS-CoV-2-negative patient pool.

a EQA, external quality assessment; VNT, virus neutralization test; ND, not determined; anti-N, antinucleocapsid; anti-S, antispike; NA, not applicable.

b Determined in undiluted sample.

c <1.0 negative; ≥1.0 positive.

d <0.8 negative; ≥0.8 positive.

e ≥1.1 positive, ≥0.8 to <1.1 borderline; <0.8 negative.

f ≥0.334 positive, <0.334 to >0.274 borderline; ≤0.274 negative.

g ≥1.1 positive, ≥0.8 to <1.1 borderline; <0.8 negative.

h ≥1.1 positive, ≥0.8 to <1.1 borderline; <0.8 negative.

i ≥1.1 positive, ≥0.8 to <1.1 borderline; <0.8 negative.

j ≥0.216 positive, <0.216 to >0.177 borderline; ≤0.177 negative.

Several immunoassays were used for detection of anti-SARS-CoV-2-specific antibodies, including the Elecsys anti-SARS-CoV-2 N and Elecsys anti-SARS-CoV-2 S tests (Roche, Germany), the anti-SARS-CoV-2 IgG and anti-SARS-CoV-2 IgA ELISAs (Euroimmun, Germany), and the EDI novel coronavirus COVID-19 IgG ELISA and EDI novel coronavirus COVID-19 IgM ELISA (Epitope Diagnostics). All tests were performed according to the manufacturers’ instructions, with the recommended cutoffs, after assay verification according to ISO 15189, and in agreement with the guidance document of the American Society for Microbiology (9).

The virus microneutralization test (VNT) was performed at the biosafety level 3 containment laboratory of the Bundeswehr Institute of Microbiology, as described previously (16). Serial dilutions from 1:10 to 1:80 of heat-inactivated serum samples were mixed with the same volume of the virus stock solution containing 100 tissue culture infectious dose 50 (TCID50) of the SARS-CoV-2 strain 2019 MUC-IMB-1. The titer of each serum sample was the highest dilution that completely neutralized the challenge dose of SARS-CoV-2. The concentration of the virus stock was also verified by back titration in each test plate.

The sample characterization results received from the program organizers’ laboratories prior to sample dispatch are summarized in Table 1; more detailed information is provided in the supplemental material. After approval of all test results by the scheme organizers and closure of the registration period, samples were dispatched to the participants.

### Statistical analysis.

Results from three distributions of the EQA scheme for anti-SARS-CoV-2 antibody detection were analyzed. The results of the data analysis are presented as descriptive statistics including sensitivity, specificity, and 95% confidence intervals (95% CI). For determination of the error rate for anti-SARS-CoV-2 IgG, anti-SARS-CoV-2 IgA, and anti-SARS-CoV-2 IgM testing, only the results reported for the respective antibody class were considered. For the method-specific error rate, only results from those laboratories using this particular method were evaluated. All statistical analyses and graph plotting were performed using R version 3.6.3 (https://www.r-project.org).

## RESULTS

### Participation.

**FIG 1:**
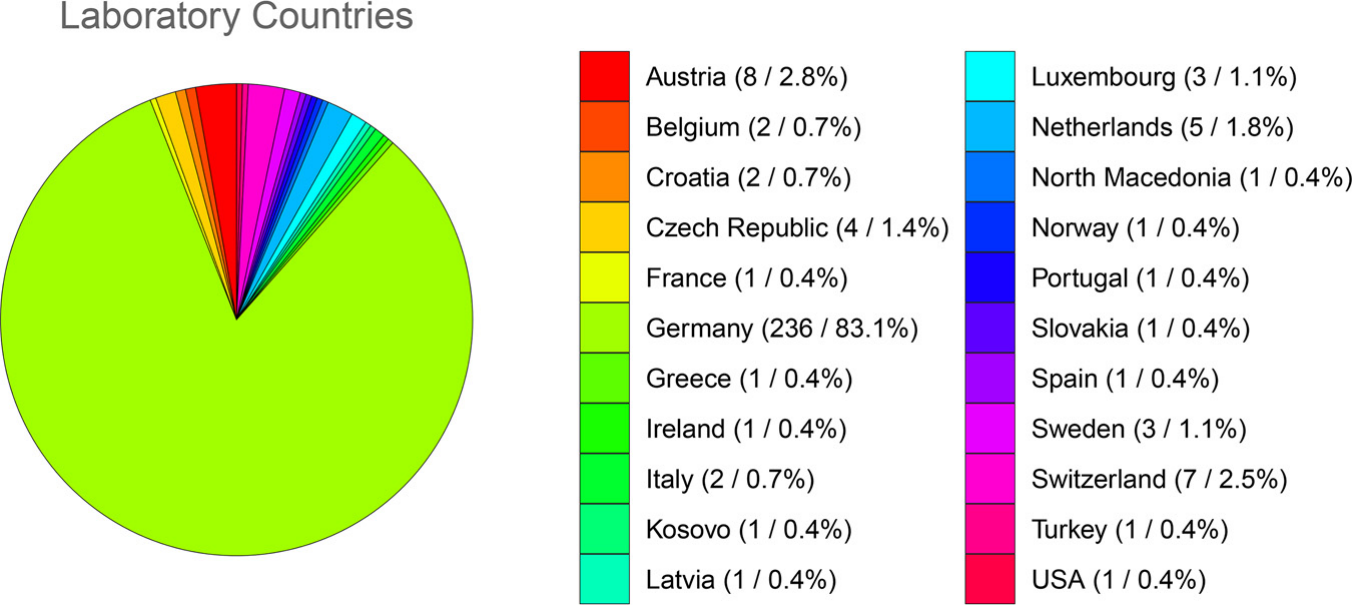
Participating laboratories per country. The pie chart depicts the total number of participating laboratories per country in the three distribution rounds of the EQA scheme for anti-SARS-CoV-2 antibody detection offered by the RfB. Each laboratory was counted as one regardless of the frequency of participation. The absolute number of laboratories and the percentage are shown (*n* = xx/%).

A total of 284 laboratories from 22 countries participated in the three open distribution rounds (EQA 1, EQA 2, EQA 3) of this EQA scheme. The majority of laboratories were from Germany (*n* = 236); one fifth were from other European countries (Fig. 1). Anti-SARS-CoV-2 IgG antibody detection was offered in each of the three distribution rounds, whereas anti-SARS-CoV-2 IgM testing was provided only in the first round, succeeded by anti-SARS-CoV-2 IgA testing in the two subsequent rounds. For anti-SARS-CoV-2 IgG testing, the number of participating laboratories increased steadily with each distribution round (182 laboratories in EQA 2 compared to 170 laboratories in EQA 1, 7.05%; 201 laboratories in EQA 3 compared to 182 laboratories in EQA 2, 10.44%). Notably, the number of laboratories participating in anti-SARS-CoV-2 IgA testing remained unchanged.

### Scope and immunoassays.

A total of 3,744 results were reported for the 12 different EQA samples provided. A total of 170, 182, and 201 laboratories participated in the three distribution rounds. For anti-SARS-CoV-2 IgG detection, totals of 992, 976, and 1,036 results were submitted in each EQA round. For anti-SARS-CoV-2 IgM, participants returned 248 in EQA 1. For anti-SARS-CoV-2 IgA, 244 and 248 results were returned in EQA 2 and EQA 3, respectively.

**FIG 2:**
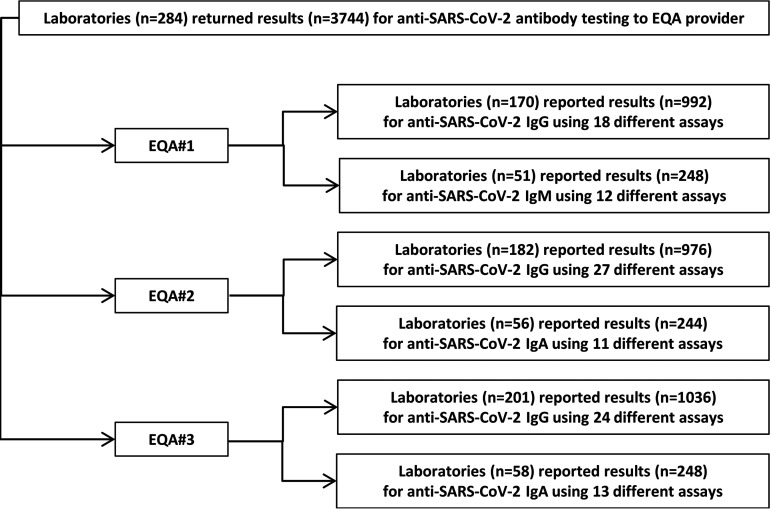
EQA design and scope. The flow diagram displays the EQA design and scope. A total of 284 laboratories returned a total of 3,733 results from testing of the 12 different EQA samples provided in three distribution rounds (EQA 1, EQA 2, and EQA 3) of the EQA scheme for anti-SARS-CoV-2 antibody detection. The laboratories could choose to participate in anti-SARS-CoV-2 IgG and/or IgM or IgA detection. The number of participating laboratories, the number of results reported, and the number of different immunoassays used by the participating laboratories are displayed for all three analytes.

For analysis of anti-SARS-CoV-2 IgG, 45.8% (78/170) of laboratories reported results for two different test systems in EQA 1. This decreased to 34.1% (62/182) in EQA 2, and a further decrease to 28.8% (58/201) was observed in EQA 3. However, the number of laboratories reporting results for two different immunoassays was substantially lower for anti-SARS-CoV-2 IgM (21.6%) and IgA detection (8.9% EQA 2 and 6.9% EQA 3). As laboratories were allowed to submit results for two different assays, the total number of participating laboratories and the number of data sets for each of the three analytes (anti-SARS-CoV-2 IgG, IgA, IgM) might differ. In this report, results are evaluated per submitted data set and for each immunoassay separately. Fig. 2 provides an overview of the EQA design and scope.

All laboratories reported the use of commercially available test systems for anti-SARS-CoV-2 antibody detection. The number of immunoassays used by the participants for anti-SARS-CoV-2 IgG testing increased from 18 different test systems in EQA 1 to 24 in EQA 3 (Table 2). In total, 29 different immunoassays were used for anti-SARS-CoV-2 IgG detection, with 9/29 having a Food and Drug Administration (FDA) EUA approval and 16/29 being CE-IVD (Conformité Européene–*In Vitro* Diagnostic Medical Device) certified. The six most frequently used commercial assays for anti-SARS-CoV-2 IgG detection were from Roche (26%), Euroimmun AG (25.4%), DiaSorin SpA (15%), Abbott Laboratories (8.3%), Epitope Diagnostics (3.1%), and Siemens Healthineers (2.1%). For anti-SARS-CoV-2 IgA and IgM detection, none of the assays used were FDA EUA approved, while 6/14 and 5/11, respectively, were CE certified.

**TABLE 2:** Participation and success rate per EQA scheme and analyte

EQA scheme no.^*a*^	Analyte	Laboratories participating (*n*)	Assays used (*n*)	Labs reporting results for 2 assays (*n*, %)	Total results submitted (*n*)	Laboratories reporting correct/conditionally correct results (*n*/%)	Laboratories reporting incorrect results (*n*/%)
1	IgG	170	18	78 (45.9%)	992	122/71.8%	48/28.2%
	IgM	51	12	11 (17.7%)	248	32/62.7%	12/37.3%
2	IgG	182	27	62 (34.1%)	976	175/96.2%	7/3.8%
	IgA	56	11	5 (8.2%)	244	25/44.6%	31/55.4%
3	IgG	201	24	58 (22.4%)	1,036	187/93.0%	14/7.0%
	IgA	58	13	4 (6.5%)	248	41/70.7%	17/29.3%

a EQA, external quality assessment.

### Success rate and sample-specific error rate.

The overall proficiency was evaluated based on the above-mentioned criteria. The target value of each EQA sample and the results reported by the participants for each sample are summarized in Table 3. Target values were assigned by the scheme organizer after detailed evaluation of the clinical information, qPCR, VNT, and immunoassay results by a panel of experts. A detailed explanation for each sample is provided in the supplemental material. For all antibody classes, results had to be reported by the participants as positive, negative, or borderline (if the absolute results were within the gray zone which was either specified by the assay manufacturer or determined by the respective laboratories) for anti-SARS-CoV-2 antibodies. Borderline results were considered inappropriate unless otherwise indicated, e.g., for sera with antibody titers near the detection limit of different immunoassays. Specifically, for cutoff samples 1 and 4, borderline results reported for IgG were considered conditionally correct, and for cutoff samples 5 and 10, all results were considered conditionally correct for IgG due to the heterogeneity of reported results, the lack of reference material and methods, and the lack of a threshold for clinically relevant antibody titers.

**TABLE 3:** Results of anti-SARS-CoV-2 antibody testing^*a*^

			No. of results submitted to RfB that were:			Error rate data	
Sample no.	EQA scheme no.	Target value	Positive (*n*)	Borderline (*n*)	Negative (*n*)	Total results reported (*n*)	Results evaluated as incorrect (*n*/%)
IgG							
1	1	Positive^*b*^	164	38	46	248	46/18.6%
2	1	Positive	237	0	11	248	11/4.4 %
3	1	Negative	4	0	244	248	4/1.6%
4	1	Negative^*b*^	6	26	216	248	6/2.4%
5	2	Positive^*c*^	90	19	135	244	0/0%
6	2	Negative	1	0	243	244	1/0.4%
7	2	Negative	1	1	242	244	2/0.8%
8	2	Positive	239	1	4	244	5/2.1%
9	3	Positive	247	0	12	259	12/4.6%
10	3	Positive^*c*^	74	50	135	259	0/0%
11	3	Positive	251	0	8	259	8/3.1%
12	3	Negative	2	0	257	259	2/0.8%
IgA							
5	2	Negative	0	7	54	61	7/11.5%
6	2	Negative	1	0	60	61	1/1.6%
7	2	Negative	9	11	41	61	20/32.8%
8	2	Positive	49	1	11	61	12/12.7%
9	3	Positive	46	0	16	62	16/25.8%
10	3	Positive	43	0	19	62	19/30.7%
11	3	Positive	47	0	15	62	15/24.2%
12	3	Negative	1	0	61	62	1/1.6%
IgM							
1	1	Negative	9	4	49	62	13/20.9%
2	1	Negative	4	4	54	62	8/12.9%
3	1	Negative	3	2	57	62	5/8.1%
4	1	Negative	5	3	54	62	8/12.9%

a RfB, Reference Institute for Bioanalytics; EQA, external quality assessment.

b For these samples, borderline results were considered conditionally correct.

c For these samples, all submitted results were considered conditionally correct.

During the scheme, the number of laboratories succeeding increased from 71.8% in EQA 1 to 93% in EQA 3 for anti-SARS-CoV-2 IgG. A comparable success rate increase was noticed for anti-SARS-CoV-2 IgA analysis (44.6% EQA 2 to 70.7% EQA 3), although the overall performance was substantially lower than that determined for IgG (Table 2).

**FIG 3:**
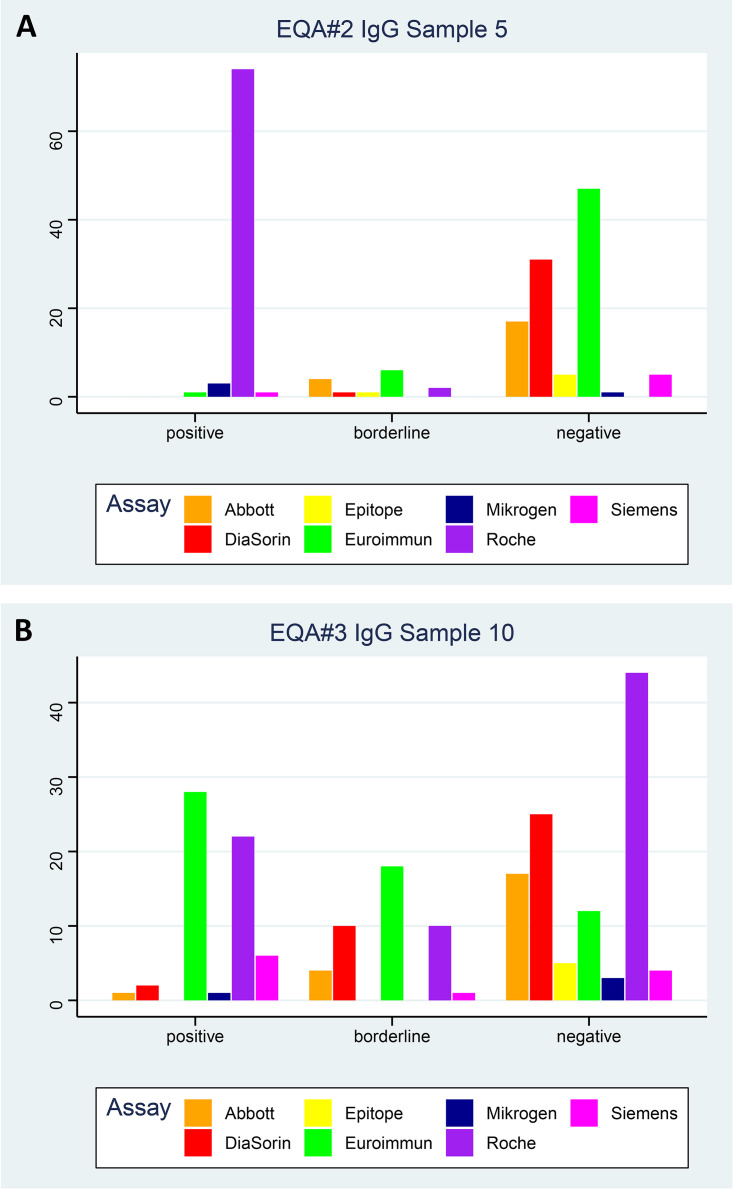
Evaluation of cutoff samples. The number of positive, borderline, and negative results submitted by the participating laboratories for the seven most frequently used commercially available assays are depicted for sample 5 in EQA 2 (A) and for sample 10 in EQA 3 (B).

For anti-SARS-CoV-2 IgG, error rates of 6.75% (67/992), 0.82% (8/976), and 2.1% (22/1036) were found in the three distribution rounds. In detail, samples 3, 4, 6, 7, and 12 were negative-control samples from a single patient, a negative patient pool, or a positive patient without detectable antibodies (sample 4). For the negative samples, 15/1243 results were determined inaccurately, resulting in a diagnostic specificity of 98.79% (95% CI, 98.02% to 99.32%). It is important to note that borderline results reported for sample 4 were considered conditionally correct. This sample was obtained from a SARS-CoV-2 PCR-positive patient without detectable antibodies at the time of first blood draw. However, the antibody levels increased over time but always remained below the respective cutoffs of the immunoassays used by the reference institutions and negative in the VNT. As 82 false-negative results were reported, a diagnostic sensitivity of 95.34% (95% CI, 94.25% to 96.28%) could be determined. In detail, for samples 1, 2, 8, 9, and 11, anti-SARS-CoV-2 IgG antibodies were detected with neutralizing activity detected in the VNT (neutralizing antibody titer ranging from 1:40 to 1:10). As sample 1 was prepared by diluting a strong positive serum to an anti-SARS-CoV-2 IgG titer near the assay detection limit, borderline results were considered conditionally correct. For these samples, 82/1,258 results were false-negative, resulting in a diagnostic sensitivity of 93.48%. However, for the two positive samples (5 and 10) with antibodies near the assay detection limit and no neutralizing antibodies detected in the VNT, very heterogeneous results were reported, as illustrated in Fig. 3. Due to the lack of reference material and tests and the heterogeneity of reported results for samples 5 and 10, borderline and negative results were considered conditionally correct and thus appropriate to receive a certificate. If only positive and borderline results for these two samples were considered accurate and negative results considered false-negative, this would have resulted in a diagnostic sensitivity of 46.32%. Strikingly, for sample 5, almost all participants using the Roche Elecsys anti-SARS-CoV-2 test obtained positive results, while the majority of results reported for other test systems were negative (Fig. 3A). In the case of sample 10, approximately the same number of positive, borderline, and negative test results were reported by the participants, regardless of the test method used. A total of 57 laboratories reported results for two different test systems for sample 10, with 31 reporting identical results for both assays and 26 reporting divergent results (Fig. 3B).

For anti-SARS-CoV-2 IgA, error rates of 16.4% (40/244) in EQA 2 and 20.6% (51/248) in EQA 3 were determined. For the four negative samples provided (5 to 7 and 12), 29/245 results reported were inaccurate, leading to a diagnostic specificity of 88.16% (95% CI, 83.44% to 91.93%). The diagnostic sensitivity was 74.90% (95% CI, 69.01% to 80.18%), with 62/247 false-negative results reported for samples 8, 9, 10, and 11.

For anti-SARS-CoV-2 IgM, 34/248 submitted results were inaccurate (error rate 13.7%). Evaluation revealed a diagnostic specificity of 86.29% (95% CI, 81.37% to 90.32%), with 34 false-positive results reported by the participants. As only negative-control samples were provided, the diagnostic sensitivity could not be determined.

### Test-specific diagnostic performance.

The diagnostic performance for anti-SARS-CoV-2 IgG, IgA, and IgM detection was evaluated for each of the assays used by the participants, and results are provided in Table 4. Diagnostic sensitivities and specificities with their respective 95% CI were also calculated. For some of the assays, diagnostic specificity and sensitivity of 100% were determined. However, these cases have a wide estimated 95% CI due to the limited number of results submitted for these test systems. For anti-SARS-CoV-2 IgG, a reliable estimation of the diagnostic test performance could be calculated for at least 6 assays. Here, substantial differences between the individual manufacturers became apparent. Overall, Abbott, Euroimmun, and Roche demonstrated the best performance, followed by DiaSorin ahead of Epitope and Virotech Diagnostics. Of note, the number of false-negative results was substantial lower in the second two EQA schemes than in the first, particularly in EQA 3/20, indicating a general improvement of the diagnostic performance. For anti-SARS-CoV-2 IgA detection, reliable test performance results were obtained for the assay from Epitope, which was used by more than 90% of participants. This had a diagnostic sensitivity of 98.22% and a diagnostic specificity of 84.92%. For anti-SARS-CoV-2 IgM, the diagnostic performances of all assays remained below the requirements for diagnostic tests.

**TABLE 4:** Assay-specific diagnostic performance^*a*^

Assay	True positive (*n*)	False negative (*n*)	True negative (*n*)	False positive (*n*)	Sensitivity (95% CI)	Specificity (95% CI)
IgG						
Abbott	145	1	102	0	99.32 (96.24–99.98)	100 (96.45–100)
Euroimmun	431	7	319	3	98.40 (96.73–99.36)	99.07 (97.30–99.81)
Ortho Clinical Diagnostic/Johnson & Johnson	3	0	1	0	100 (29.24–100)	100 (2.50–100)
Becton Dickinson	2	0	2	0	100 (15.81–100)	100 (15.81–100)
Beckmann Coulter	9	0	7	0	100 (66.37–100)	100 (59.04–100)
BioMerieux	5	0	3	0	100 (47.82–100)	100 (29.24–100)
Eppendorf	1	1	2	0	50.0 (1.26–98.47)	100 (15.81–100)
Siemens Healthineers	3	0	1	0	100 (29.24–100)	100 (2.50–100)
Roche	459	7	312	2	98.5 (96.93–99.39)	99.36 (97.72–99.92)
DRG	6	0	6	0	100 (54.07–100)	100 (54.07–100)
Siemens Healthineers (Advia)	42	0	22	0	100 (91.59–100)	100 (84.56–100)
Mediagnost	5	0	3	0	100 (47.82–100)	100 (29.24–100)
Immundiagnostik	7	3	9	1	70.0 (34.75–93.33)	90.0 (55.50–99.75)
Medipan Diagnostika	4	0	4	0	100 (39.76–100)	100 (39.76–100)
DiaSorin	241	20	183	4	92.34 (88.41–95.26)	97.86 (94.61–99.41)
Epitope Diagnostics	40	11	41	0	78.43 (64.68–88.71)	100 (91.4–100)
Virotech Diagnostics	20	4	20	0	83.33 (62.62–95.26)	100 (83.16–100)
Other	134	18	100	4	88.16 (81.93–92.83)	96.15 (90.44–98.94)
MöLab	5	1	5	1	83.33 (35.88–99.58)	83.33 (35.88–99.58)
GA (Generic Assay)	12	0	8	0	100 (73.54–100)	100 (63.06–100)
AESKU.Diagnostics	6	0	2	0	100 (54.07–100)	100 (15.81–100)
R-Biopharm	5	0	3	0	100 (47.82–100)	100 (29.24–100)
nal von minden GmbH	15	3	14	0	83.33 (58.58–96.42)	100 (76.84–100)
Microgen	26	0	18	0	100 (86.77–100)	100 (81.47–100)
Novatech	5	3	8	0	62.5 (24.49–91.48)	100 (63.06–100)
Viramed Biotech	11	0	9	0	100 (71.51–100)	100 (66.37–100)
Willi Fox	6	1	5	0	85.71 (42.13–99.64)	100 (47.82–100)
SchBo	2	0	2	0	100 (15.81–100)	100 (15.81–100)
Bühlmann	5	0	3	0	100 (47.82–100)	100 (29.24–100)
Siemens Healthineers (Vista)	3	0	1	0	100 (29.24–100)	100 (2.50–100)
Virion/Serion	6	0	2	0	100 (54.07–100)	100 (15.81–100)
Advanced Instruments	2	2	4	0	50.0 (6.76–93.24)	100 (39.76–100)
Vircell	13	0	7	0	100 (75.29–100)	100 (59.04–100)
EQA 2/20	439	57	486	10	88.51 (85.37–91.18)	97.98 (96.32–99.03)
EQA 3/20	483	5	485	3	98.98 (97.63–99.67)	99.39 (98.21–99.87)
EQA 4/20	757	20	257	2	97.43 (96.05–98.42)	99.23 (97.24–99.91)
Total	1,679	82	1,228	15	95.34 (94.25–96.28)	98.79 (98.02–99.32)
IgA						
Abbott	3	3	2	0	50.0 (11.81–88.19)	100 (15.81–100)
Euroimmun	166	3	152	27	98.22 (94.90–99.63)	84.92 (78.82–89.82)
Becton Dickinson	0	1	3	0	0 (0.00–97.50)	100 (29.24–100)
BioMerieux	2	1	1	0	66.67 (9.43–99.16)	100 (2.50–100)
DRG	0	1	3	0	0 (0.00–97.50)	100 (29.24–100)
Mediagnost	4	0	4	0	100 (39.76–100)	100 (39.76–100)
Virotech Diagnostics	0	9	11	0	0 (0.00–33.63)	100 (71.51–100)
Other	4	15	9	0	21.05 (6.05–45.57)	100 (66.37–100)
AESKU.Diagnostics	0	3	1	0	0 (0.00–60.76)	100 (2.50–100)
R-Biopharm	0	4	4	0	0 (0.00–60.24)	100 (39.76–100)
nal von minden GmbH	2	1	1	0	66.67 (9.43–99.16)	100 (2.50–100)
Microgen	0	4	4	0	0 (0.00–60.24)	100 (39.76–100)
Novatech	0	5	7	0	0 (0.00–52.18)	100 (59.04–100)
Viramed Biotech	0	6	9	1	0 (0.00–45.93)	90.00 (55.50–99.75)
Vircell	4	6	5	1	40.0 (12.16–73.76)	83.33 (35.88–99.58)
EQA 2/20	ND	ND	ND	ND	ND	ND
EQA 3/20	49	12	155	28	80.33 (68.16–89.40)	84.70 (78.65–89.59)
EQA 4/20	136	50	61	1	73.12 (66.14–79.34)	98.39 (91.34–99.96)
Total	185	62	216	29	74.90 (69.01–80.18)	88.16 (83.44–91.93)
IgM						
Epitope Diagnostics	0	0	68	0	NA	100 (94.72–100)
nal von minden GmbH	0	0	10	2	NA	83.33 (51.59–97.91)
Advanced Instruments	0	0	8	0	NA	100 (63.06–100)
Other	0	0	70	26	NA	72.92 (62.89–81.48)
Immundiagnostik	0	0	12	0	NA	100 (73.54–100)
Virotech Diagnostics	0	0	15	1	NA	93.75 (69.77–99.84)
Euroimmun	0	0	3	1	NA	75.0 (19.41–99.37)
Novatech	0	0	12	0	NA	100 (73.54–100)
GA-Generic Assay	0	0	4	0	NA	100 (39.76–100)
MöLab	0	0	6	2	NA	75.0 (34.91–96.81)
Viramed Biotech	0	0	4	0	NA	100 (39.76–100)
Willi Fox	0	0	2	2	NA	50.0 (6.76–93.24)
EQA 2/20	0	0	214	34	NA	86.29 (81.37–90.32)
EQA 3/20	ND	ND	ND	ND	ND	ND
EQA 4/20	ND	ND	ND	ND	ND	ND
Total	0	0	214	34	NA	86.29 (81.37–90.32)

a EQA, external quality assessment.

## DISCUSSION

This EQA was conducted between May and November 2020 to gain insights into the landscape of current anti-SARS-CoV-2 serological diagnostics at a European level, to assess the performance of and identify potential weaknesses in the proficiency of both laboratories and test systems, and finally, to provide recommendations for future improvements. In general, EQA is a key strategy for achieving harmonization, and thus a high standard, of diagnostic procedures (14, 18). In the case of COVID-19, this is particularly important, as diagnostic results do not only affect a single individual but influence health, social, economic, and political decisions worldwide (19).

In this EQA scheme, samples were dispatched at ambient temperature, as in other serological PT offered by the RfB and in accordance with the results of the stability testing performed. This scheme was a category IV EQA survey in which commutable samples were provided, allowing determination of the measurement performance of individual laboratories and assessment of the uniformity between laboratories and measurement procedures (13). Evaluation of the three distribution rounds of this first European PT for anti-SARS-CoV-2 antibody detection revealed several issues relevant to the quality of serological diagnostics as well as to their improvement.

First, the participation of 170 laboratories in the first distribution round and the increasing number of participants in the following schemes prove that serological anti-SARS-CoV-2 diagnostics is widely implemented and offered by numerous laboratories. The most frequently tested antibody class is IgG. This makes diagnostic sense considering the current state of science and the results of this EQA scheme. Anti-SARS-CoV-2 IgM detection was replaced by IgA in the second distribution round due to the poor diagnostic performance of the IgM tests, with a specificity of 86.3%. This is in line with available studies (16, 20–22). The diagnostic performance of anti-SARS-CoV-2 IgA detection, with a specificity of 88.2% and a sensitivity of 74.9%, does not meet the diagnostic requirements of a sensitivity of ≥90% and a specificity of ≥95%, as initially requested by FDA, or a sensitivity of ≥90% and a specificity of ≥98%, as required by the European Commission (23–25). These results support the recommendation of the Center of Disease Control and Prevention (CDC) that IgA testing currently should not be used (8).

Second, evaluation of this scheme revealed a very heterogeneous diagnostic landscape for anti-SARS-CoV-2 IgG detection, with 31.0% of tests used being FDA approved and 55.2% CE certified. Thus, a substantial proportion of laboratories are currently using uncertified kits for clinical diagnostics, which might be explained by limited availability of consumables (26). However, this affects the quality of the diagnostics: FDA-approved tests show an overall sensitivity of 97.2% and a specificity of 99.1%, CE-certified ones a sensitivity of 96.5% and a specificity of 99.2%, while the sensitivity and specificity of noncertified tests are 88.5% and 96.7%, respectively. Given these substantial differences in test performance between certified and noncertified tests, the impact of results on management of the ongoing pandemic, and the short time laboratories have for a proper validation/verification of tests, there should at least be a strong recommendation to use certified tests for standard care by professional societies or by regulatory guidance (as it is the case in the United States, for example).

Third, the overall diagnostic performance of anti-SARS-CoV-2 IgG detection is adequate, with a diagnostic sensitivity of 95.3% and a specificity of 98.8%. Detailed evaluation revealed considerable differences between the test systems used, with those from Abbott, Euroimmun, and Roche showing the best results. These test differences are consistent with the results of published studies (5, 27–33). For example, Favresse et al. reported a diagnostic sensitivity of 95.4% for the Euroimmun ELISA, followed by 92% for the Roche Elecsys test and 88.5% for DiaSorin (31). However, Harritshoj et al. recently demonstrated a substantially inferior performance when comparing 16 different serological SARS-CoV-2 immunoassays in 16 laboratories. Here, a sensitivity of 90.0% was reported for Abbott, 78.0% for Euroimmun, and 92.7% for Roche (32). These differences can most likely be explained by sample selection or, more specifically, the number of samples included from patients with asymptomatic SARS-CoV-2 infection. In general, only test systems with a good diagnostic performance should be used in a clinical setting. Detailed test performance data are provided within this report.

Fourth, the CDC recommends that detection of anti-SARS-CoV-2 antibodies be limited to test systems with a specificity greater than 99.5% (8). However, none of the test systems used by the 284 laboratories participating in this EQA program met this requirement, with the exception of Abbott’s SARS-CoV-2 IgG assay. The Roche Elecsys anti-SARS-CoV-2 test and the anti-SARS-CoV-2 IgG ELISA from Euroimmun narrowly missed this requirement, with specificities of 99.4% and 99.1%, respectively. For some assays, the limited number of reported results could affect results, and thus poor performance of individual laboratories could cause such low specificities. However, at least for tests with more than 100 reported results for negative samples, specificity can be reliably assessed. Another explanation could be the limited number and selection of negative samples dispatched in this EQA. However, the number of samples is sufficient for an EQA and all samples were from negative tested participants without clinical symptoms within the last months. Therefore, the most likely explanation for the lower specificities revealed by this EQA compared to those from data from test providers and some published studies is the interlaboratory variability, which is usually evident only in EQAs with hundreds of participants. Taking this observation into consideration, strategies to increase pretest probability and limit anti-SARS-CoV-2 serological testing to specific patient cohorts with increased risk should be pursued, particularly in low-prevalence settings.

Fifth, although the overall diagnostic performance of anti-SARS-CoV-2 IgG detection was acceptable, there were considerable differences in performance depending on the samples’ antibody titers. While the diagnostic sensitivity for samples from PCR-positive patients with antibodies detected in the VNT (which has an analytical sensitivity lower than that of common immunoassays) was 93.5%, the diagnostic sensitivity for cutoff samples from PCR-positive specimens in which neutralizing antibodies were not detected decreased to 46.3%. The low diagnostic sensitivity for cutoff samples may be due to the time course of antibody development, with sensitivity increasing with antibody level. In general, antibodies become detectable at approximately 1 to 2 weeks postinfection, peak approximately 30 to 35 days after symptom onset, and have a longevity of several months (34, 35). The cutoff samples provided in the various distribution rounds were either prepared by diluting a strong positive sample to the assay detection limit or obtained no earlier than 30 days after qPCR-based diagnosis. Therefore, it is unlikely that the low sensitivity for these samples is influenced by antibody dynamics. In this context, it is worth mentioning that Mulchandani et al. have already described an overestimation of the test performance of anti-SARS-CoV-2 antibody detection reported in the literature, explained by the restriction to PCR-confirmed cases, leading to a spectrum bias. They reported a drop in the sensitivity from 94.2% among PCR-confirmed cases of SARS-CoV-2 to 84.7% among unselected populations (28). This general overestimation of test performance should be considered when interpreting patient results for clinical decision making.

Sixth, detailed evaluation of the two cutoff samples yielded additional findings. For sample 5, all positive results were obtained almost exclusively with the Roche Elecsys anti-SARS-CoV-2 assay, which detects antibodies targeting the nucleocapsid protein (36). That detection of the nucleocapsid protein is more sensitive has been described previously (37) and is consistent with the fact that the highest diagnostic sensitivity in the literature is reported for the Roche assay (28, 31). For sample 10, the interlaboratory variability was tremendous regardless of the assay used. This highlights the high measurement uncertainty and the lack of uniformity between laboratories and measurement procedures. To improve the diagnostic quality of anti-SARS-CoV-2 antibody tests and to achieve a harmonization of test results, optimization of cutoffs is urgently needed. Laboratories are currently forced to validate appropriate cutoffs or gray areas independently in order to guarantee high diagnostic quality. One principal way to determine the analytical cutoff is to measure samples with known concentrations (ideally prepared by dilution of reference material) and determine the minimum antibody titer that can be reliably detected. Specifically, the coefficient of variation at the assay detection limit or for cutoff samples should be as low as possible, e.g., <5%. The diagnostic cutoff should be defined by ROC curve analysis and needs to fulfill general requirements, e.g., a diagnostic specificity of >99.5% and a sensitivity of >90% in case of SARS-CoV-2. Regarding the definition of appropriate cutoffs, another point has to be considered. To date, the relationship between antibody titer and protective immunity has not been fully elucidated, with initial reports suggesting a specific threshold required for a sufficient immune response (38). Hence, further studies are warranted to define adequate, clinically relevant cutoffs. This, along with appropriate reference material and EQA results, will help assay manufacturers to determine reliable cutoffs for anti-SARS-CoV-2 antibody detection in the future.

Seventh, another possibility to improve the diagnostic performance is orthogonal testing. Here, positive test results are confirmed by a distinct immunoassay targeting a different antigen (7). This strategy is also recommended by the CDC (8). However, the number of laboratories reporting results for two different assays decreased from 46% to 22% during this scheme. Furthermore, even with an orthogonal testing strategy, a significant number of questionable or incorrect results would still be reported. In the case of sample 10, this strategy would have failed in 50% of cases. Thus, orthogonal testing could help to improve results, especially for cutoff samples, but further technical improvements are still needed.

A limitation of this EQA scheme is the lack of standardized reference material and methods for determination of anti-SARS-CoV-2 antibodies. Therefore, the assignment of target values could only be based on clinical information, results of different immunoassays, and VNT, with sensitivity lower than that of common serological assays and thus not suitable to reliably evaluate cutoff samples. Hence, the assessment was done by an expert panel, which could cause bias in the results. In particular, because all results for the two cutoff samples were considered correct for IgG but not for IgA, an overestimation of the IgG assay performance is likely, as illustrated for example by the low number of false-negatives for IgG in EQA 3/20. The limited number of samples provided in this scheme is another limitation of this study. However, the number of samples dispatched within each scheme is identical to that of other serological EQAs offered by the RfB and other EQA providers. A prerequisite for the evaluation of EQA results is that negative, positive, and borderline samples (to challenge assay performance) are provided. This scheme fulfills all requirements for a category IV EQA, which allows to evaluate the individual performance of each participating laboratory in general and in comparison to a peer, to determine interlaboratory variability to assess reproducibility, and finally to evaluate the standardization and harmonization of results relative to the participants’ results (13). Due to the lack of reference methods/material and as no samples were sent in duplicate, individual laboratory variability, absolute accuracy of each laboratory, and absolute assay performance compared to a reference method cannot be assessed.

In conclusion, this first EQA for anti-SARS-CoV-2 antibody detection was conducted by the RfB to assess the current quality of serological testing in the ongoing COVID-19 pandemic. The high number of participants proves that this diagnostic is established firmly in clinical care. The PT showed a heterogeneous diagnostic landscape, with the test systems used having divergent diagnostic performances. In particular, the results for cutoff samples demonstrate the lack of harmonization of measurement procedures. As serological testing will continue to gain attention in the context of vaccination, it is of upmost importance to improve the diagnostic performance. Among the recommendations made based on the results of this EQA is the restriction to anti-SARS-CoV-2 IgG detection, both for diagnostic purposes and for future iterations of this EQA scheme, due to the lack of reference material and reference methods and the heterogeneity of results for IgA detection. Certified assays with a high diagnostic performance should be used and conscientiously verified prior to clinical use, preferably by using reference material if available. If available, this should be used for proficiency testing in addition to clinical samples. Furthermore, the strategies of increasing pretest probability and orthogonal testing should be followed. Most importantly, appropriate cutoffs must be defined in order to harmonize testing procedures and thus obtain reproducible and reliable results for clinical decision-making.
