# Supplementary material for: Assessing the Quality of Serological Testing in the COVID-19 Pandemic: Results of a European External Quality Assessment (EQA) Scheme for Anti-SARS-CoV-2 Antibody Detection
Source: J Clin Microbiol. 2021 Aug 18;59(9):e00559-21. doi: 10.1128/JCM.00559-21 (PMC8373014; doi:10.1128/JCM.00559-21)
Supplement: Supplemental file 1 — Text S1 (supplemental clinical information) and legend of Fig. S1. Download JCM.00559-21-s0001.pdf, PDF file, 0.7 MB [file jcm.00559-21-s0001.pdf]

Supplemental material

Assessing the quality of serological testing in the CoViD-19 pandemic: results of a European external quality assessment (EQA) scheme for anti-SARS-CoV-2 antibody detection

Volker Ast[1], Victor Costina[1], Romy Eichner[1], Anna Bode[2], Sihem Aida[1], Catharina Gerhards[1], Margot Thiaucourt[1], Gerhard Dobler[3,4], Wolf-Jochen Geilenkeuser[2], Roman Wölfel[3,4], Michael Neumaier[1], Verena Haselmann[1]

Inventory of supplemental material

Supplemental information: Clinical characteristics EQA samples

Supplemental Figure

Supplemental Information

Clinical characteristics of EQA samples

Medical history and evaluation of sample #1:

28-year-old male SARS-CoV-2 positive tested, symptomatic (shortness of breath, loss of smell and taste, night sweats and muscle aches) outpatient. He has a medical history of exertion asthma and hay fever. No information about blood group was provided.

Blood sampling was performed 34 days after qRT-PCR confirmation of SARS-CoV-2 infection. Anti-SARS-CoV-2 IgG antibodies with neutralizing activity, but no IgM antibodies were detected by different immunoassays performed by the reference institute. Neutralizing antibody titer 1:40 in SARS-CoV-2 Micro-NAT in undiluted sample, but <1:10 in diluted sample (2:1 total to sample) that was shipped in this EQA.

Definition and brief explanation of assigned target values for anti-SARS-CoV-2 IgG: As this sample was obtained by diluting of a strong positive sample (NAT 1:40) to an anti-SARS-CoV-2 IgG titer near the assay detection limit, borderline results were additionally given as conditionally correct.

Definition and brief explanation of assigned target values for anti-SARS-CoV-2 IgM: positive and borderline test results were considered as incorrect as no anti-SARS-COV-2 IgM antibodies were detected neither in the undiluted nor the diluted samples by the reference institute. The IgM antibody dynamics with a peak at around two weeks after symptom onset with rapidly diminishing titers supports this decision, particularly in a strong diluted sample.

Medical history and evaluation of sample #2:

56-year-old female SARS-CoV-2 positive tested, asymptomatic outpatient. She has a medical history of Hashimoto thyroiditis and is currently on treatment with L-Thyroxin. No information about blood group was provided.

Blood sampling was performed 39 days after qRT-PCR confirmation of SARS-CoV-2 infection. Anti-SARS-CoV-2 IgG antibodies were detected by different immunoassays performed by the reference institute with neutralizing activity. Neutralizing antibodies against SARS-CoV-2 isolated MUC-IMB-01 detected in Micro-NAT (1:10). No anti-SARS-CoV-2 IgM antibodies detected.

Definition and brief explanation of assigned target values for anti-SARS-CoV-2 IgG: Negative and borderline results were considered as inaccurate as antibodies were detectable in the Micro-NAT knowing to have a lower sensitivity than immunoassays.

Definition and brief explanation of assigned target values for anti-SARS-CoV-2 IgM: Positive and borderline test results were considered as incorrect as no anti-SARS-COV-2 IgM antibodies were detected by the reference institute. The IgM antibody dynamics with a peak at around two weeks after symptom onset with rapidly diminishing titers supports this decision as does the number of laboratories reporting positive or borderline results.

Medical history and evaluation of sample #3:

54-year-old female SARS-CoV-2 negative control patient. No history of respiratory infection or common cold within the last six month. Further medical history was unremarkable. Blood group A rhesus negative.

No anti-SARS-CoV-2 IgG or IgM antibodies were detected by the reference institute.

Definition and brief explanation of assigned target values for anti-SARS-CoV-2 IgG: positive and borderline results were considered inaccurate as the sample was obtained from a negative-tested participant without clinical symptoms within the last months.

Definition and brief explanation of assigned target values for anti-SARS-CoV-2 IgM: positive and borderline results were considered inaccurate as the sample was obtained from a negative-tested participant without clinical symptoms within the last months.

<u>Medical history and evaluation of sample #4</u>:

35-year-old female SARS-CoV-2 positive tested, oligo symptomatic (anosmia, altered taste) outpatient. Medical history was non-contributory. Blood group AB rhesus positive.

Blood sampling was performed 26 days after qRT-PCR confirmation of SARS-CoV-2 infection. No anti-SARS-CoV-2 IgG or IgM antibodies were detected by the reference institute. Neutralizing antibodies against SARS-CoV-2 isolated MUC-IMB-01 were not detected in Micro-NAT (<1:10).

Definition and brief explanation of assigned target values for anti-SARS-CoV-2 IgG: positive results were considered as inaccurate as no antibodies were detectable in the Micro-NAT and in the immunoassays used by the reference institute. However, in the long-time follow-up of the patient, a seroconversion was observed with the values increasing, but still being under the LOD (limit of detection) of the respective assays. Therefore, borderline results were accepted as conditionally correct as other test systems might be more sensitive.

Definition and brief explanation of assigned target values for anti-SARS-CoV-2 IgM: Positive and borderline test results were considered as incorrect as no anti-SARS-COV-2 IgM antibodies were detected by the reference institute. The IgM antibody dynamics with a peak

at around days xx-xx with rapidly diminishing titers supports this decision as does the fact that IgG antibodies were not detectable or stayed below assay LOD over time.

Medical history and evaluation of sample #5:

27-year-old female SARS-CoV-2 positive tested, oligo symptomatic (fever, night sweats, altered taste) outpatient. She has a medical history of psoriasis. Blood group 0 rhesus positive.

Blood sampling was performed 37 days after qRT-PCR confirmation of SARS-CoV-2 infection. Anti-SARS-CoV-2 IgG antibodies without neutralizing activity, but no IgA antibodies were detected by different immunoassays performed by the reference institute. Neutralizing antibody titer <1:10 in SARS-CoV-2 Micro-NAT of the diluted sample (2:1 total to sample) that was shipped in this EQA scheme. As the sample was diluted near to the assay detection limit, indeterminate results were given as correct and negative test results as conditionally correct.

Definition and brief explanation of assigned target values for anti-SARS-CoV-2 IgG: As the sample was diluted near to the assay detection limit, borderline results were given as correct and negative test results as conditionally correct. This decision was based on the fact that without a positive Micro-NAT and quantitative assays, it was not possible to safely assure the presence of antibodies, particularly in a clinical relevant titer. Thus, all results were considered as correct or as conditionally correct. An alternative would have been to exclude this sample from evaluation of this scheme. The RfB decided to report the results as this highlights the variability of assay results and thus the need for cut-off harmonization. Furthermore, we agreed on issuing certificates regardless of the results reported for this

sample, as this is unlikely to be a laboratory mistake or a shortcoming of one single test, but a limitation/shortcoming of currently available immunoassays. A detailed evaluation of this sample is also provided in the result section of the main manuscript.

Definition and brief explanation of assigned target values for anti-SARS-CoV-2 IgA: positive and borderline results were considered inaccurate as no antibodies were detected by the reference institute and this is also supported by the number of laboratories reporting positive/borderline results.

Medical history and evaluation of sample #6:

41-year-old male SARS-CoV-2 negative control patient. No history of respiratory infection or common cold within the last six month. Further medical history was unremarkable. Blood group 0 rhesus positive.

No anti-SARS-CoV-2 IgG or IgA antibodies were detected by the reference institute.

Definition and brief explanation of assigned target values for anti-SARS-CoV-2 IgG: positive and borderline results were considered inaccurate as the sample was obtained from a negative-tested participant without clinical symptoms within the last months and no antibodies detected by the reference institute.

Definition and brief explanation of assigned target values for anti-SARS-CoV-2 IgA: positive and borderline results were considered inaccurate as the sample was obtained from a negative-tested participant without clinical symptoms within the last months and no antibodies detected by the reference institute.

Medical history and evaluation of sample #7:

SARS-CoV-2 negative control patient pool from eleven different donors. All donors did not have a history of respiratory infection or common cold within the last six month and were SARS-CoV-2 negative tested by qRT-PCR.

No anti-SARS-CoV-2 IgG or IgA antibodies were detected by the reference institute.

Definition and brief explanation of assigned target values for anti-SARS-CoV-2 IgG: positive and borderline results were considered inaccurate as the sample was obtained from eleven negative-tested participants without clinical symptoms within the last months and no antibodies detected by the reference institute.

Definition and brief explanation of assigned target values for anti-SARS-CoV-2 IgA: positive and borderline results were considered inaccurate as the sample was obtained from eleven negative-tested participants without clinical symptoms within the last months. All patients were negative for anti-SARS-CoV-2 IgG. Although IgA antibodies were detected by the reference institute, this was considered as cross-reactivity.

Medical history and evaluation of sample #8:

40-year-old male SARS-CoV-2 positive tested, symptomatic inpatient treated for 21 days at the intensive care unit (at first presentation fever, within course of disease he developed pneumonia and an acute respiratory distress syndrome requiring intensive mechanical ventilation). He has a medical history of allergic asthma and mononucleosis. No information about blood group was provided.

Blood sampling was performed 59 days after qRT-PCR confirmation of SARS-CoV-2 infection. Anti-SARS-CoV-2 IgG and IgA antibodies were detected by different immunoassays performed by the reference institute with neutralizing activity. Neutralizing antibodies

against SARS-CoV-2 isolated MUC-IMB-01 were detected in Micro-NAT (1:20) in the diluted sample (2:1 total to sample) that was distributed in this EQA scheme.

Definition and brief explanation of assigned target values for anti-SARS-CoV-2 IgG: Negative and borderline results were considered as inaccurate as antibodies were detectable in the Micro-NAT knowing to have a lower sensitivity than immunoassays.

Definition and brief explanation of assigned target values for anti-SARS-CoV-2 IgA: Negative and borderline results were considered as inaccurate as antibodies were detectable in the Micro-NAT knowing to have a lower sensitivity than immunoassays.

<u>Medical history and evaluation of sample #9</u>:

52-year-old female SARS-CoV-2 positive tested, symptomatic (fever, muscle aches, shortness of breath, loss of smell and taste) outpatient. Further medical history was noncontributory. Blood group 0 rhesus positive.

Blood sampling was performed 85 days after qRT-PCR confirmation of SARS-CoV-2 infection. Anti-SARS-CoV-2 IgG and IgA antibodies were detected by different test systems performed by the reference institute with neutralizing activity. Neutralizing antibody titer 1:20 in SARS-CoV-2 Micro-NAT in diluted sample (1.14:1 total to sample) that was shipped in this EQA.

Definition and brief explanation of assigned target values for anti-SARS-CoV-2 IgG: Negative and borderline results were considered as inaccurate as antibodies were detectable in the Micro-NAT knowing to have a lower sensitivity than immunoassays.

Definition and brief explanation of assigned target values for anti-SARS-CoV-2 IgA: Negative and borderline results were considered as inaccurate as antibodies were detectable by the

reference institute and in the Micro-NAT knowing to have a lower sensitivity than immunoassays.

Medical history and evaluation of sample #10:

28-year-old female SARS-CoV-2 positive tested, symptomatic (shortness of breath, loss of smell and taste, muscle aches) outpatient. She has a suspected Hashimoto thyroiditis and is currently on treatment with L-Thyroxin. No information about blood group was provided.

Blood sampling was performed 32 days after qRT-PCR confirmation of SARS-CoV-2 infection.

Anti-SARS-CoV-2 IgG and IgA antibodies were detected by different test systems performed by the reference institute without neutralizing activity. Neutralizing antibody titer <1:10 in SARS-CoV-2 Micro-NAT of the undiluted sample dispatched in the EQA scheme.

Definition and brief explanation of assigned target values for anti-SARS-CoV-2 IgG: positive and borderline results were considered as correct as this was a cut-off sample provided without neutralizing antibodies detected. Due to the high number of laboratories reporting negative results, these were considered as conditionally correct. Comparable to sample #5 all participants received a certificate regardless of the result reported for this sample. The reason this sample was not excluded from the scheme is identical to sample #5.

Definition and brief explanation of assigned target values for anti-SARS-CoV-2 IgA: In difference to IgG, the majority of laboratories reported positive results comparable to those obtained before sample dispatch by the reference institute. Accordingly, there was no need to consider all results as conditionally correct.

Medical history and evaluation of sample #11:

44-year-old male SARS-CoV-2 positive tested, symptomatic (fever, night sweats, diarrhea, shortness of breath, loss of smell and taste, muscle aches) outpatient. The further medical history was unremarkable. No information about blood group was provided.

Blood sampling was performed 47 days after qRT-PCR confirmation of SARS-CoV-2 infection.

Anti-SARS-CoV-2 IgG and IgA antibodies were detected by different test systems performed by the reference institute with neutralizing activity. Neutralizing antibody titer 1:40 in SARS-CoV-2 Micro-NAT in diluted sample (1.14:1 total to sample) that was shipped in this EQA.

Definition and brief explanation of assigned target values for anti-SARS-CoV-2 IgG: Negative and borderline results were considered as inaccurate as antibodies were detectable in the Micro-NAT knowing to have a lower sensitivity than immunoassays.

Definition and brief explanation of assigned target values for anti-SARS-CoV-2 IgA: Negative and borderline results were considered as inaccurate as antibodies were detectable by the reference institute and in the Micro-NAT knowing to have a lower sensitivity than immunoassays.

Medical history and evaluation of sample #12:

SARS-CoV-2 negative control patient pool from six different donors. All donors did not have a history of respiratory infection or common cold within the last six month and were SARS-CoV-2 negative tested by qRT-PCR.

No anti-SARS-CoV-2 IgG or IgA antibodies were detected by the reference institute.

Definition and brief explanation of assigned target values for anti-SARS-CoV-2 IgG: positive and borderline results were considered inaccurate as the sample was obtained from a

negative-tested participant without clinical symptoms within the last months and no antibodies detected by the reference institute.

Definition and brief explanation of assigned target values for anti-SARS-CoV-2 IgA: positive and borderline results were considered inaccurate as the sample was obtained from a negative-tested participant without clinical symptoms within the last months and no antibodies detected by the reference institute.

Supplemental Figures:

Supplemental Figure 1: Workflow of EQA sample preparation by the RfB

The schematic depicts the workflow of EQA sample preparation and characterization performed by the reference institute before sample dispatch. Firstly, blood was withdrawn from voluntary donors who had given their written informed consent (1). Secondly, serum blood samples were centrifuged after appropriate clotting time at 2000 *g* for 10 minutes at 18°C, and were pooled (2). Thirdly, each serum stock was aliquoted á 600µl (3). Fourthly, three aliquots out of each stock were used for anti-SARS-CoV-2 antibody testing and detection of neutralizing antibodies by the reference institute (4). After anti-SARS-CoV-2 ELISA-based antibody detection and virus neutralization testing, samples were shipped to the participating laboratories (5). Laboratories were asked to use their standard operation procedure for detection of anti-SARS-CoV-2 IgG, IgM or IgA antibodies for each of the samples provided (7). Finally, results were returned by the laboratories to the EQA provider (Reference Institute for Bioanalytics) (8).
