# Supplementary figures and images for: Assessing the Quality of Serological Testing in the COVID-19 Pandemic: Results of a European External Quality Assessment (EQA) Scheme for Anti-SARS-CoV-2 Antibody Detection
Source: J Clin Microbiol. 2021 Aug 18;59(9):e00559-21. doi: 10.1128/JCM.00559-21 (PMC8373014; doi:10.1128/JCM.00559-21)

Supplemental Figure 1

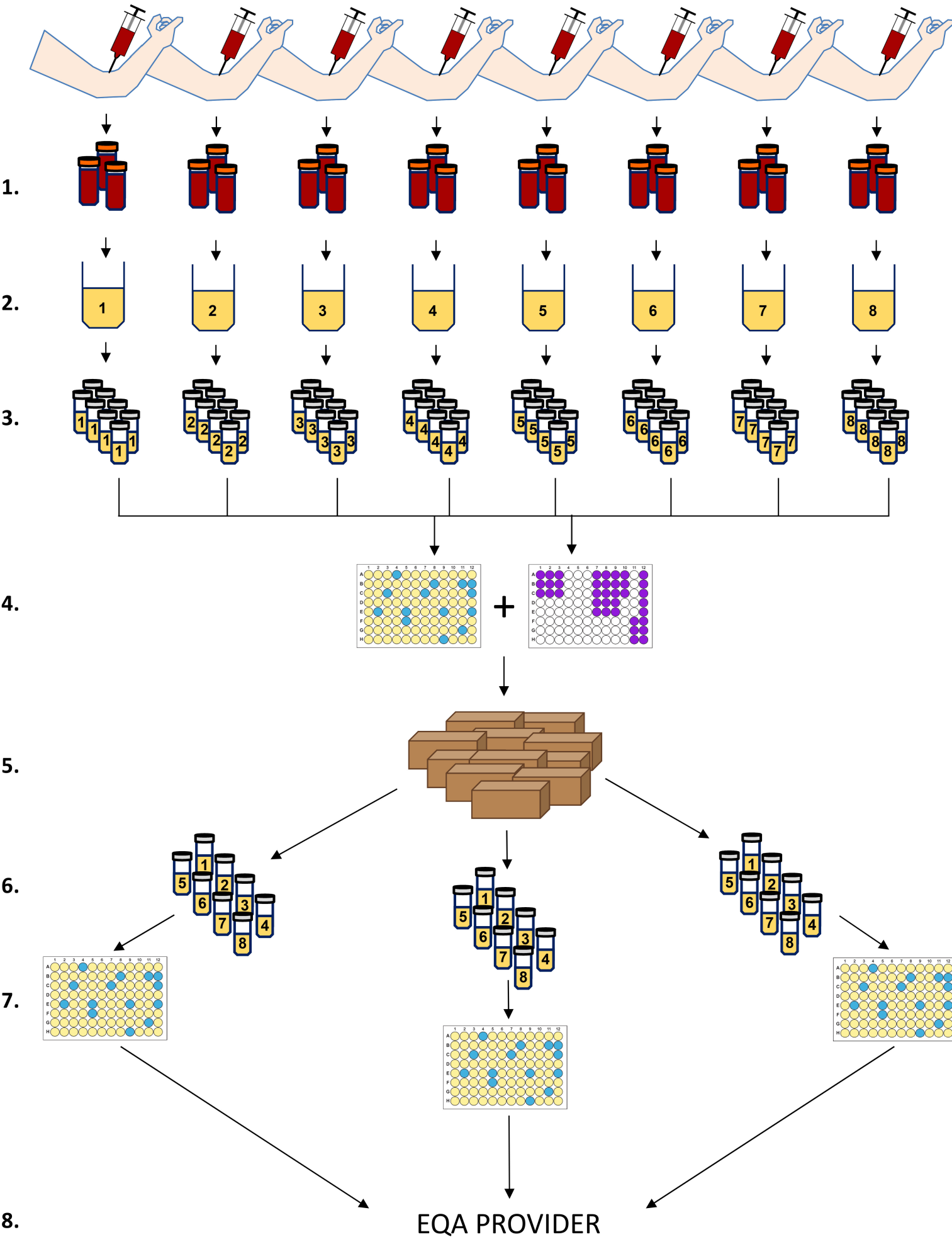

Supplement: Supplemental file 2 — Fig. S1. Download JCM.00559-21-s0002.pdf, PDF file, 4.4 MB [file jcm.00559-21-s0002.pdf]
